# Nitric Oxide (NO) Mediates the Inhibition of Form-Deprivation Myopia by Atropine in Chicks

**DOI:** 10.1038/s41598-016-0002-7

**Published:** 2016-12-05

**Authors:** Brittany J. Carr, William K. Stell

**Affiliations:** 1 Neuroscience Graduate Program, Snyder Institute for Chronic Diseases, Alberta Children’s Hospital Research Institute, Hotchkiss Brain Institute, Calgary, Alberta Canada; 2 Department of Cell Biology and Anatomy and Department of Surgery; Cumming School of Medicine, University of Calgary, Calgary, Alberta Canada

## Abstract

Myopia is the most common childhood refractive disorder. Atropine inhibits myopia progression, but its mechanism is unknown. Here, we show that myopia-prevention by atropine requires production of nitric oxide (NO). Form-deprivation myopia (FDM) was induced in week-old chicks by diffusers over the right eye (OD); the left eye (OS) remained ungoggled. On post-goggling days 1, 3, and 5, OD received intravitreally 20 µL of phosphate-buffered saline (vehicle), or vehicle plus: NO source: L-arginine (L-Arg, 60–6,000 nmol) or sodium nitroprusside (SNP, 10–1,000 nmol); atropine (240 nmol); NO inhibitors: L-NIO or L-NMMA (6 nmol); negative controls: D-Arg (10 µmol) or D-NMMA (6 nmol); or atropine plus L-NIO, L-NMMA, or D-NMMA; OS received vehicle. On day 6 post-goggling, refractive error, axial length, equatorial diameter, and wet weight were measured. Vehicle-injected goggled eyes developed significant FDM. This was inhibited by L-Arg (ED50 = 400 nmol) or SNP (ED50 = 20 nmol), but not D-Arg. Higher-dose SNP, but not L-Arg, was toxic to retina/RPE. Atropine inhibited FDM as expected; adding NOS-inhibitors (L-NIO, L-NMMA) to atropine inhibited this effect dose-dependently, but adding D-NMMA did not. Equatorial diameter, wet weight, and metrics of control eyes were not affected by any treatment. In summary, intraocular NO inhibits myopia dose-dependently and is obligatory for inhibition of myopia by atropine.

## Introduction

Myopia (near- or short-sightedness) is the refractive error in which images of objects at infinity are focussed in front of the photoreceptors, causing blurred distance vision. It is the most common childhood vision disorder, affecting up to 35% of North American children, and its prevalence is increasing worldwide^1^. This refractive error can be corrected by lenses or surgery, but there is no generally accepted way to prevent the onset or progression of myopia. Common optical corrections fail to address the underlying defect (excessive axial elongation), and therefore reduce neither the risk of visual impairment due to comorbidities^2^ nor the associated increases in health care costs.

One strategy for combating childhood myopia is to administer growth-inhibiting drugs. Despite numerous clinical trials of other agents, only atropine has become widely accepted; therefore, it is used to combat myopia in countries such as Singapore and Taiwan, where prevalence is epidemic^3^. This broad-spectrum competitive inhibitor of acetylcholine-binding at muscarinic acetylcholine receptors (mAChR) inhibits myopia development in some children when applied topically^4^. However, at the most commonly used dose (1%) it produces unacceptable side effects, including photophobia, paralysis of accommodation, and allergic reactions^5^. Additionally, it is not effective in all children, and a “rebound effect” may occur when treatment is terminated^6^. Atropine is also effective against myopia in avian and mammalian animal models, in which it mainly inhibits the exaggerated axial elongation that occurs during myopia development. Other mAChR antagonists that do not have as severe side effects as atropine have been investigated in humans^7, 8^ and animals^9–11^, however, they generally have no effect^9^. Two exceptions are pirenzepine and tropicamide, but while their therapeutic effects are statistically significant, their effects are clinically insignificant^3^. Current literature leaves a large gap in our understanding of the potential role of mAChR antagonists in regulation of eye size; there is consensus that the mechanism underlying atropine inhibition of myopia does not rely on paralysis of accommodation^12^, but the rest remains largely unknown. Because of atropine’s decades-long popularity as a myopia-prevention tool, it is important to understand the mechanism by which it prevents excessive eye growth. This should allow us to further our understanding of the underlying mechanisms of emmetropia, and to identify possible alternative targets through which myopia can be prevented, without the negative side-effects of atropine.

One possible therapeutic alternative might be something that activates the production of nitric oxide (NO). NO is considered to be a “light-adaptive” signalling molecule; it is known to mediate some light-adaptive changes in the retina^13–16^, and its synthesis and release are increased by intense or intermittent (flickering) illumination^17, 18^. When applied to the retina, NO donors mimic the adaptational effects of increased illumination^19^, while inhibitors of nitric oxide synthase (NOS) – the enzyme that generates NO from L-arginine – mimic the functional effects of decreased illumination in light-adapted chicks^20^. Recently, increased environmental illumination has been reported to protect against myopia in animals^21, 22^ and children^23, 24^, and it has been reported that NOS-inhibitors block the prevention of experimentally-induced form-deprivation myopia (FDM) normally elicited by daily periods of unobstructed vision^25^. Taking this evidence into consideration, we tested the hypothesis that increased ocular nitric oxide synthesis is (i) sufficient to prevent FDM on its own, and (ii) necessary for atropine-mediated myopia prevention in the chick. A preliminary report of our findings was presented previously (Carr B, *et al*. *IOVS* 2013; 54: E-Abstract 3677).

## Results

### Normal Ocular Growth and Myopia-Development after Application of Form-Diffuser Goggles

**Figure 1:**
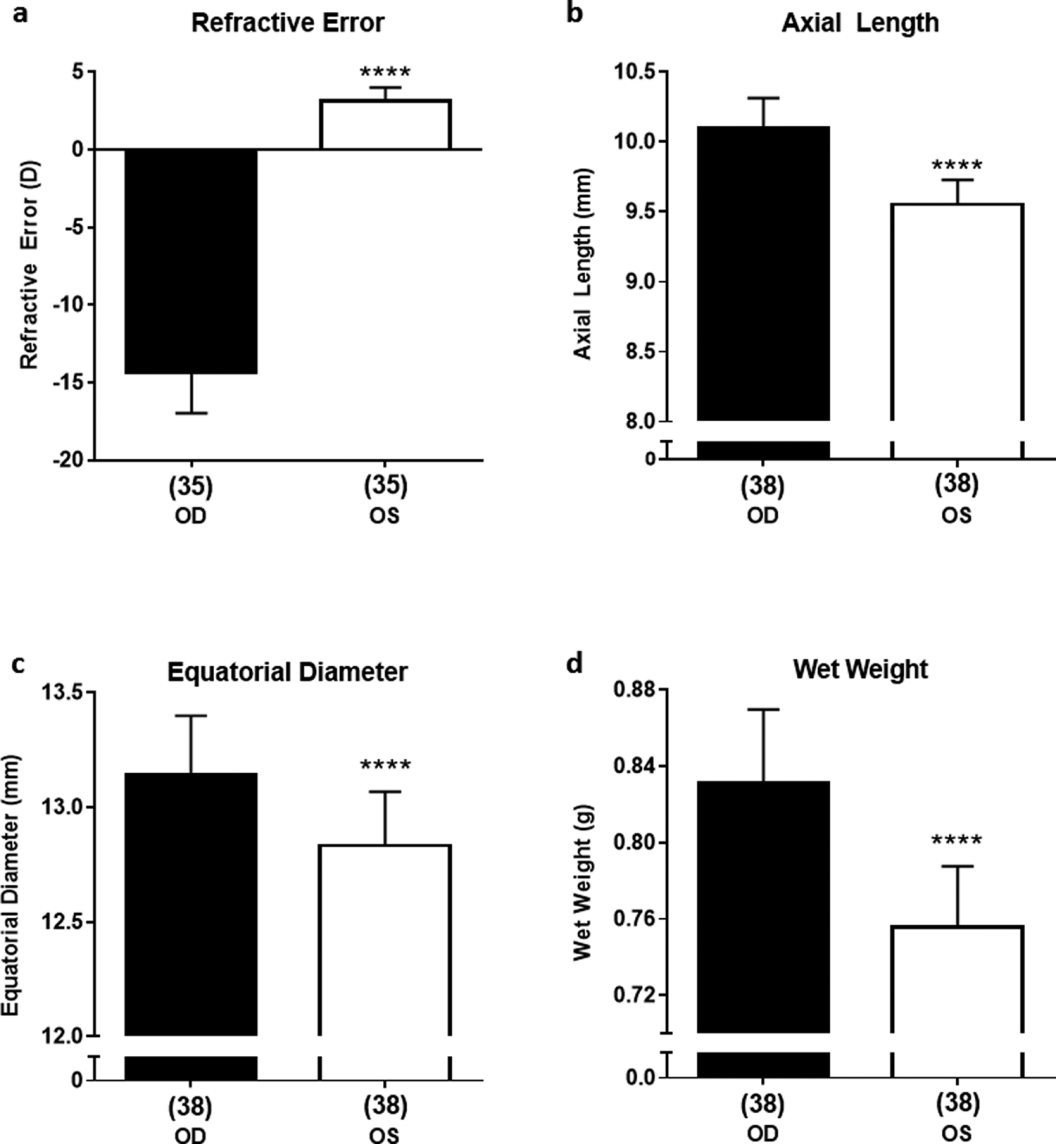
Refractive and biometric interocular differences between goggled (form-deprived right eye: OD) and non-goggled (open left eye: OS) eyes, after injecting phosphate-buffered saline (PBS) 3 times, at 48-hr intervals. Monocular form-deprivation of chicks in this study caused the expected responses: a large negative shift in refractive error (**a**), and modest but highly significant increases in total axial length (**b**), circular-equivalent equatorial diameter (**c**), and wet weight (**d**), in the goggled eyes. Data are represented as absolute mean ± standard deviation. ***Statistics***: ****p < 0.0001, unpaired Student’s *t*-test; sample sizes (n) are denoted in brackets below each column.

Data are represented as absolute values ± SD. Control eyes (open, vehicle) from all treatment groups exhibited a mean hyperopic refractive error of 3.2 ± 0.8 D and axial length of 9.55 ± 0.18 mm at the end of the treatment period. There was no significant difference between these parameters of control eyes in any of the treatment groups (One-Way ANOVA, p = 0.8807); therefore, they were used as same-animal standards for comparison of effects in treated eyes, minimizing any confounding effects of inter-individual differences. Goggled eyes that received saline injections developed significant myopia, exhibiting increases in negative refractive error (RE), axial length (AL), equatorial diameter (ED), and wet weight (WW) compared to those parameters in contralateral control eyes (RE: −14.36 ± 2.7 D vs. 3.2 ± 0.8 D; AL: 10.10 ± 0.22 mm vs. 9.55 ± 0.18 mm; ED: 13.14 ± 0.26 mm vs. 12.83 ± 0.23 mm; WW: 0.831 ± 0.039 g vs. 0.756 ± 0.032 g; p < 0.0001, unpaired *t*-test, two-tailed; n = 35–38). This verified that our goggles induced significant axial myopia and increased eye size in these animals, even with repeated intravitreal injections (Fig. 1a–d).

### Inhibition of Form-Deprivation Myopia by NO-Sources

**Figure 2:**
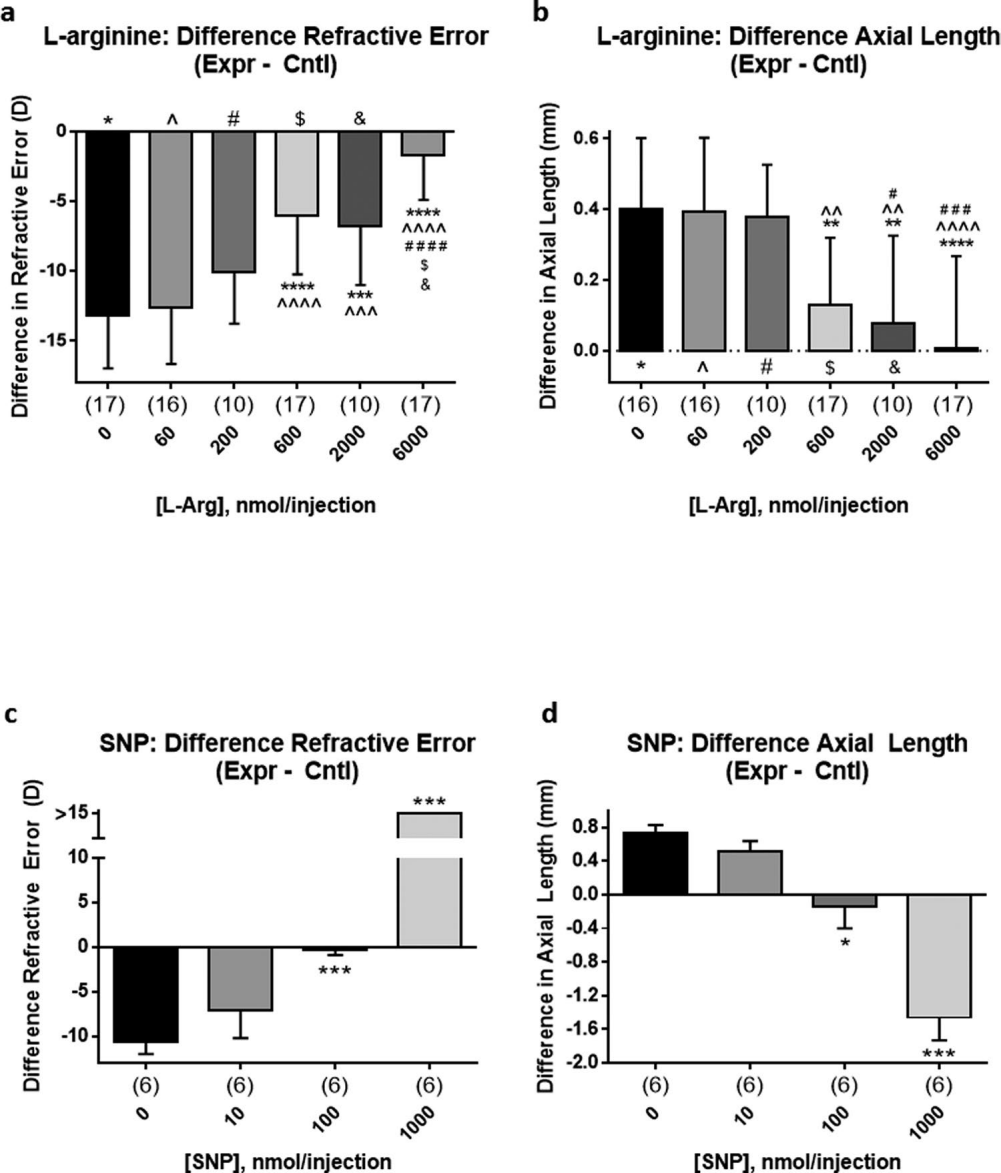
The effects of exogenous nitric oxide (NO), delivered intravitreally as NOS-substrate (L-arginine; L-Arg) or NO-donor (sodium nitroprusside; SNP), on form-deprivation myopia in chicks; doses represent the molar amounts of drug injected, per injection, 3 times at 48-hr intervals. L-Arg (**a,b**) and SNP (**c,d**) dose-dependently inhibited the development of myopic refractive error (**a,c**) and excessive axial elongation (**b,d**) in goggled chick eyes; the refractions and sizes of control eyes were not affected by drugs delivered to the goggled eyes. ***Symbols***: asterisk (*): comparison to effect of PBS-treatment; caret (^): comparison to 60 nmol; pound (#): comparison to 200 nmol; dollar ($): comparison to 600 nmol; ampersand (&): comparison to 2000 nmol. ***Statistics***: ^••••^p < 0.0001, ^•••^p < 0.001, ^••^p < 0.01, ^•^p < 0.05; L-Arg: One-Way ANOVA + Tukey’s post-hoc; SNP: Kruskal-Wallis + Dunn’s post-hoc. Data are represented as the means of the difference in values for the experimental eye minus those for the control eye, ±SD; sample sizes (n) are denoted in brackets below each column.

Data are represented as the mean difference between values for the experimental eye minus those for the control eye ±SD. L-Arg (pH 7), at 60–6,000 nmol (maximum vitreal concentrations 0.3–30 mM), decreased the effects of the diffuser goggle in a dose-dependent manner (n = 10–17), with an ED_50_ = 400 nmol (maximum vitreal concentration 2 mM) (Fig. 2a,b). At a dose of 10 µmoles, D-Arg – which does not serve as a source of NO – did not alter the growth and refraction of normal or form-deprived eyes (Supplementary Fig. S1, n = 5–6). To validate further that myopia inhibition by L-arginine is mediated by an increase in NO, we tested the effect of simultaneous injection of 6 nmol L-NMMA with 600 nmol of L-Arg; addition of L-NMMA resulted in a complete blockade of myopia inhibition by L-Arg (Supplementary Fig. S1, n = 5–10). Sodium nitroprusside (SNP), at 10–1,000 nmol (maximum vitreal concentrations 0.05–5 mM), decreased myopia development dose-dependently, with ED_50_ = 20 nmol (maximum vitreal concentration 100 µM; n = 6) (Fig. 2c,d). However, eyes treated with the highest dose of SNP (1,000 nmol) were extremely hyperopic and had significantly shortened axial lengths; this is typically an indicator of drug toxicity, and histological examination after toluidine blue staining and immunolabelling revealed that retinas from these highly shortened eyes were grossly abnormal.

### Effects of L-Arg and SNP on Retinal Integrity

#### Toludine blue

**Figure 3:**
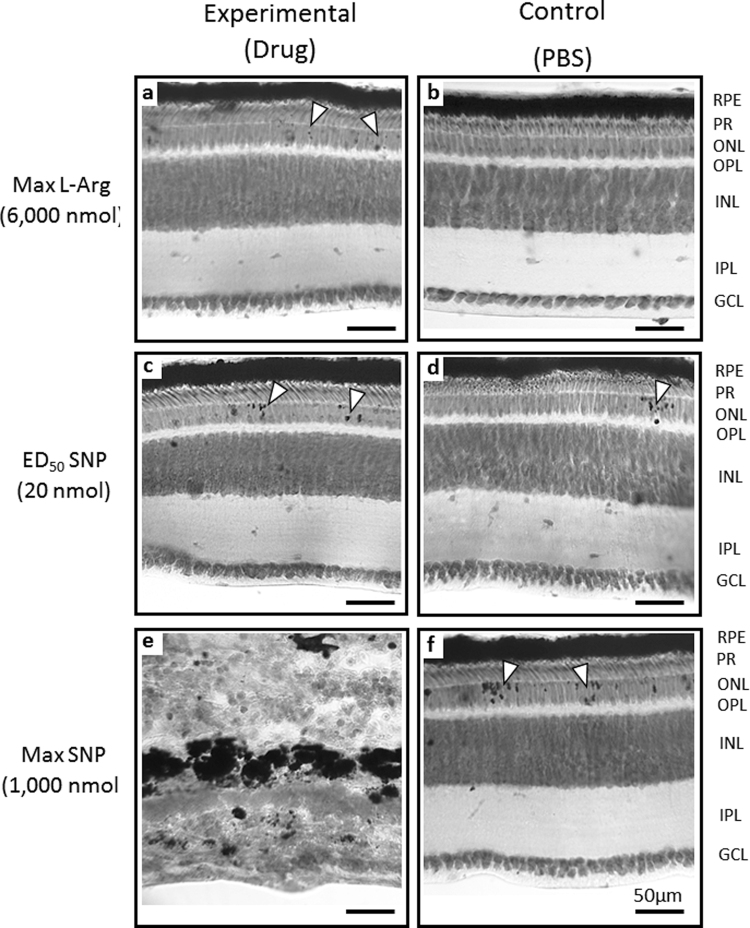
Transmission light micrographs of Toluidine blue-stained retinas treated with L-Arg and SNP, and their contralateral PBS-treated controls. There was no obvious damage to retinal or RPE structure in eyes treated with either the ED_50_ (Supplementary Fig. S2) or the maximum dose of L-Arg (**a**), but small pigment deposits were present in the ONL (▽) that were not seen in the PBS-only control (**b**). At the ED_50_ of SNP, there was an increase in the small pigment deposits in the ONL (▽), in treated (**c**) and control (**d**) tissues; otherwise, the structure of the retina and RPE remained largely unaffected. At the maximum concentration of SNP, there was massive degeneration of the retina and RPE, with complete loss of the photoreceptor and ONL/OPL; INL and GCL were still detectable, but distorted (**e**). Contralateral control eyes (**f**) had a significant increase in pigment in the ONL; but the structure of the retina and RPE remained intact. ***Abbreviations:*** RPE [retinal pigment epithelium]; PR [photoreceptor inner and outer segments (bacillary layer)]; ONL [outer nuclear layer]; OPL [outer plexiform layer]; INL [inner nuclear layer]; IPL [inner plexiform layer]; GCL [ganglion cell layer]. Widefield (25x, NA 0.8). Scale bar = 50 µm.

At the ED_50_ (400 nmol) and maximum (6,000 nmol) doses, L-Arg caused no retinal damage; scattered small pigment aggregates were detected in the outer nuclear layer (ONL) at the highest dose, but not in controls (Fig. 3a,b; Supplementary Fig. S2). At ED_50_ of SNP (20 nmol), pigment aggregates were more abundant in both treated and control eyes, but retinal and RPE structure remained largely intact (Fig. 3c,d). Retina and RPE of eyes treated with the maximum dose of SNP (1,000 nmol) were severely degenerated, with complete loss of outer retinal layers and significant distortion of inner layers (Fig. 3e); ONL pigment aggregates were larger and more frequent in fellow control eyes, indicating possible contralateral-eye effects of high-dose SNP (Fig. 3f).

#### Immunolabelling

**Figure 4:**
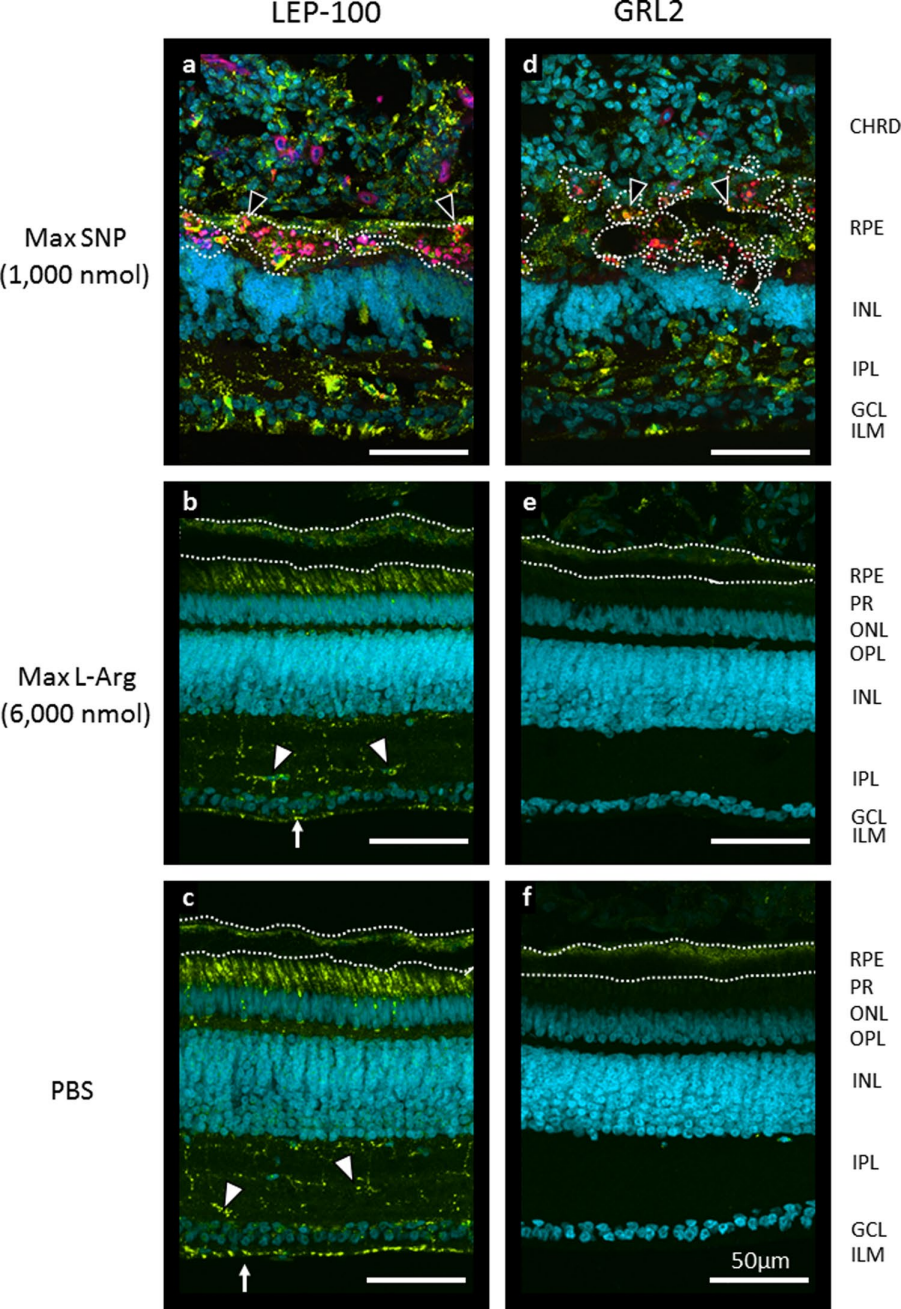
Fluorescence micrographs of retinal sections, labeled yellow-green for LEP-100 or GRL2 and teal-blue for nuclei (DAPI); RPE and pigmented structures, which we located in bright-field images, are outlined with white dotted lines. In retinas treated with the maximal dose of SNP (**a**,**d**), intense LEP-100 and GRL2 signals were found in the IPL, near the inner limiting membrane, and in the choroid; a significant amount of red autofluorescence (pseudo-colored pink) was detected in what are most likely the pigmented remnants of the RPE, some of which were co-localized with the LEP-100 and GRL2 signals (▼). In all other tissues tested, there was no significant fluorescence in the red channel, and LEP-100 and GRL2 labelling patterns did not differ according to treatment group. LEP-100 signal was present in the basal RPE, bacillary layer (PR), putative microglia/macrophage in the IPL (▽), and probable astrocytes at the internal limiting membrane () in max L-Arg- and PBS-treated retina (**b**,**c**). GRL2 signal was not detected in undamaged retina (**e**,**f**). ***Abbreviations:*** CHRD [choroid]; RPE [retinal pigment epithelium]; PR [photoreceptor inner and outer segments (bacillary layer)]; ONL [outer nuclear layer]; OPL [3outer plexiform layer]; INL [inner nuclear layer]; IPL [inner plexiform layer]; GCL [ganglion cell layer]; ILM [inner limiting membrane]. Images are maximum-intensity Z-stack projections of the entire thickness of retinal sections (12–14 µm, 1.5 µm/slice); scale bar = 50 µm.

LEP-100 (an indicator of phagocytosis) in control eyes was apparent in the basal RPE, retinal bacillary layer (photoreceptor outer and inner segments), outer plexiform layer, and putative microglia/macrophages^26^ and astrocytes in the inner retina (Fig. 4c); the activated leukocyte marker, GRL2, only weakly labeled the basal RPE (Fig. 4f). L-Arg at any dose tested, and SNP at ED_50_, caused no change in these labelling patterns (Fig. 4b,e; Supplementary Fig. S2); but, retinas heavily damaged by maximum-dose SNP were intensely labelled for LEP-100 and GRL2, and red autofluorescence was abundant in putative RPE remnants (Fig. 4a,d).

### Inhibition of Form-Deprivation Myopia by Atropine and NOS-Specific Blockade of Inhibition

**Figure 5:**
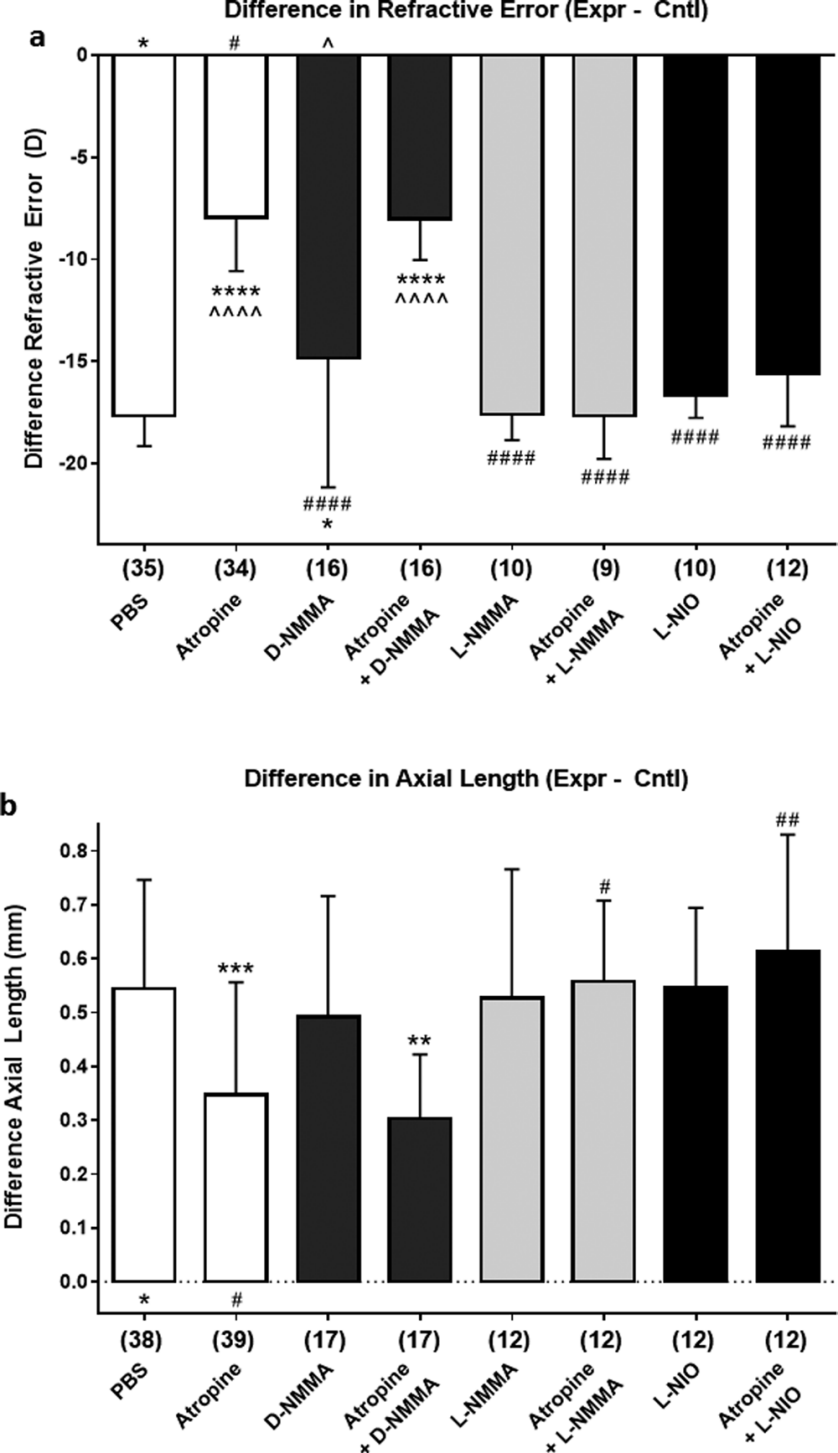
The effects of atropine (240 nmol), NOS inhibitors (6 nmol; L-NIO, L-NMMA), D-NMMA (6 nmol), and the combination of atropine + NOS inhibitors (L-NIO, L-NMMA), or atropine + D-NMMA on refractive error (**a**) and axial length (**b**); treatment schedule as in Fig. 1. ***Abbreviations:*** **L-NIO** [N^G^-(1-Iminoethyl)-L-ornithine]; **L-NMMA** [L-N^G^-monomethyl arginine]; **D-NMMA** [D-N^G^-monomethyl arginine]. ***Symbols***: asterisk (*): comparison to effect of PBS-treatment; pound (#): comparison to effect of atropine-treatment; caret (^): comparison to effect of D-NMMA-treatment. ***Statistics:*** ^••••^p < 0.0001, ^•••^p < 0.001, ^••^p < 0.01, ^•^p < 0.05; One-Way ANOVA + Tukey’s post-hoc. Data are represented as the means of the difference in values for the experimental eye minus those for the control eye, ±SD; sample sizes (n) are denoted in brackets below each column.

Data are represented as the means of the difference (d) in values for the experimental eye minus those for the control eye, ±SD. All outcomes are listed in Table 1, statistical p-values are listed in Table 2, and data are visualized in Fig. 5. Intravitreal atropine (240 nmol) significantly inhibited FD-induced refractive error and axial elongation (dRE: −8.0 ± 2.1 D vs. −17.7 ± 1.5 D, p < 0.0001, n = 34; dAL: 0.35 ± 0.2 mm vs 0.54 ± 0.20 mm, p = 0.0006, n = 39). Intravitreal injection of NOS inhibitors (6 nmol; L-NIO and L-NMMA) had no effect on the development of FDM; the dRE closely matched that of vehicle controls, and were significantly different from that of atropine-treated eyes (L-NIO, dRE: −16.0 ± 2.5 D, p < 0.0001, n = 10; L-NMMA, dRE: −15.6 ± 2.6 D, p < 0.0001, n = 10). NOS-inhibition did not affect FD-induced changes in dAL (L-NIO, dAL: 0.55 ± 0.2 mm, p = 0.054, n = 12; L-NMMA, dAL: 0.56 ± 0.2 mm, p = 0.1149, n = 12). The mean dRE of eyes injected with D-NMMA (6 nmol) was only slightly less negative than that of controls (dRE: −14.8 ± 6.3 D, p = 0.033, n = 16), but very different from that of atropine-treated eyes (p < 0.0001); the mean dAL of D-NMMA-injected eyes was not significantly different from that of atropine-treated eyes (dAL: 0.49 ± 0.2 mm; p = 0.1917, n = 17).

**Table 1:** The effects of atropine (240 nml), NOS inhibitors (6 nmol), and D-NMMA (6 nmol) on the mean difference between experimental (goggled) eyes and control (non-goggled) eyes of various eye parameters.

Treatment	dRE (D)	dAL (mm)	dED (mm)	dWW (g)
PBS	−17.7 ± 1.5	0.54 ± 0.20	0.31 ± 0.23	0.075 ± 0.033
Atropine (Atro)	−8.0 ± 2.6	0.35 ± 0.21	0.30 ± 0.16	0.087 ± 0.030
L-NIO	−16.0 ± 2.5	0.55 ± 0.15	0.33 ± 0.18	0.085 ± 0.018
Atro + L-NIO	−15.6 ± 2.6	0.61 ± 0.22	0.38 ± 0.20	0.100 ± 0.028
L-NMMA	−15.6 ± 2.6	0.56 ± 0.24	0.21 ± 0.25	0.062 ± 0.040
Atro + L-NMMA	−17.7 ± 2.1	0.56 ± 0.15	0.39 ± 0.20	0.082 ± 0.039
D-NMMA	−14.8 ± 6.3	0.49 ± 0.22	0.38 ± 0.22	0.080 ± 0.035
Atro + D-NMMA	−8.0 ± 2.0	0.30 ± 0.12	0.34 ± 0.16	0.081 ± 0.016

*dRE: difference refractive errors; dAL: difference axial lengths; dED: difference equatorial diameters; dWW: difference wet weights. Values represented as mean ± SD.

**Table 2:** Adjusted p-values (Tukey’s post hoc) for all significantly different means of the difference between eyes for refractive error (dRE) and axial lengths (dAL) from atropine-, NOS inhibitor-, and D-NMMA-treatment experiments.

Treatment Groups	dRE	dAL
PBS vs. Atropine (Atro)	<0.0001	0.0006
PBS vs. D-NMMA	0.0331	
PBS vs. Atro + D-NMMA	<0.0001	0.0012
Atro vs. D-NMMA	<0.0001	
Atro vs. L-NMMA	<0.0001	
Atro vs. Atro + L-NMMA	<0.0001	0.0321
Atro vs. L-NIO	<0.0001	
Atro vs. Atro + L-NIO	<0.0001	0.0019
D-NMMA vs. Atro + D-NMMA	<0.0001	
Atro + D-NMMA vs. L-NMMA	<0.0001	0.0173
Atro + D-NMMA vs. Atro + L-NMMA	<0.0001	
Atro + D-NMMA vs. L-NIO	<0.0001	0.0281
Atro + D-NMMA vs. Atro + L-NIO	<0.0001	0.0013

*dRE: difference refractive errors; dAL: difference axial lengths.

Simultaneous injection of NOS inhibitors with atropine interfered with the prevention of myopia (atropine + L-NIO, dRE: −15.6 ± 2.6 D, p < 0.0001, n = 12; dAL: 0.61 ± 0.2 mm, p = 0.002, n = 12; atropine + L-NMMA, dRE: −17.7 ± 2.1 D, p < 0.0001, n = 9; dAL: 0.56 ± 0.2 mm, p = 0.0321, n = 12). In contrast, D-NMMA (the biologically inactive enantiomer) had no effect on myopia-prevention by atropine; after treatment with atropine + D-NMMA, dRE and dAL were still significantly smaller than in PBS-controls (dRE: −8.05 ± 2.0 D, p < 0.0001, n = 16; dAL: 0.30 ± 0.1 mm, p = 0.0012, n = 17). No drug treatments significantly affected equatorial diameter or wet weight (Supplementary Fig. S3). To validate these results, we evaluated the effect of decreasing concentrations of L-NMMA on atropine-mediated inhibition of myopia; 60 and 600 pmol L-NMMA (n = 9–11) had no effect on atropine’s ability to prevent FDM, while 6 nmol L-NMMA (n = 7) blocked inhibition of myopia by atropine (Supplementary Fig. S4).

## Discussion

In these experiments, we tested whether nitric oxide plays a role in regulation of eye growth and atropine-mediated prevention of form-deprivation myopia. As hypothesized, atropine and NO-sources inhibited FDM in a dose-dependent manner, and NOS-inhibitors blocked the atropine-mediated inhibition of myopia. In contrast, D-Arg and D-NMMA – enantiomers that are inactive at NOS – had no effect; therefore, the blocking of myopia-prevention by NOS inhibitors is likely due to the stereospecific actions of these L-Arg analogs at NOS, rather than non-specific effects of arginine-like compounds via other molecular targets and processes^27^.

Histological examination of retinas treated with L-Arg at all doses, and SNP at ED_50_, revealed no significant damage to retina and RPE. At the maximum applied dose of SNP, however, we observed massive destruction of both tissues and dramatic changes in the associated choroid. Eye growth (specifically, size of the sclera) is controlled by retinal activity, relayed through the RPE and choroid^28^; therefore, the damage observed in these tissues likely explains the arrest of elongation, highly positive refractive error (Fig. 2c), and shortened axial length (Fig. 2d) of these SNP-treated eyes – indicating, coincidentally, that *signalling by the retina/RPE and/or the choroid constitutively promotes ocular elongation during the post-hatching period of rapid growth*. The intense labelling for LEP-100 and GRL2 in the IPL and choroid, and the appearance of red autofluorescence in RPE-choroid at the highest dose of SNP, likely indicate increased macrophage activation^26^, phagocytosis of cell debris^29^, and gliosis.

Atropine is used off-label to prevent myopia-progression and is still the only drug used widely for this purpose; nevertheless, its mechanism of action and the role of ACh in regulation of eye growth remain obscure. Muscarinic receptors *are* present in chicken eye tissues; immunohistochemistry with affinity-purified subtype-specific antibodies (cm^2^/cm^3^/cm^4^) revealed the presence of mAChRs in the chick retina, retinal pigment epithelium, choroid, and ciliary body^30^, which has been confirmed by radioligand binding^31^. However, form-deprivation (FD) does not affect mRNA or protein expression of any subtype of mAChR in chick retina^31^. FD has no effect on the concentration of acetylcholine, or its metabolite choline^32^, and ablating over 90% of choline acetyltransferase-producing amacrine cells has no effect on the eye’s ability to achieve emmetropia, nor does it impair myopia-inhibition by atropine^33^. Research on atropine-mediated prevention of myopia has been based largely on the assumption that, because atropine is a muscarinic antagonist, it prevents myopia by acting at mAChR(s). However, this has *never* been conclusively shown, and as we have argued recently^34^, many data are inconsistent with atropine-mediated prevention of myopia by competitive inhibition of ACh at retinal mAChRs. Additionally, the evidence for direct interaction between ACh and modulation of NOS in the retina is sparse and conflicting. For example, oxotremorine increases immunoreactive cGMP via mAChR M2 in salamander retina^35^, but via mAChR M1/M3 in rat^36^; and while induction of nitric oxide enhances light-evoked release of ACh from amacrine cells in the rabbit^37^, it *inhibits* high K^+^-evoked release of ACh in the rat^38^. In the chick, no studies such as these have been published.

Nitric oxide (NO) is synthesized by the enzyme nitric oxide synthase (NOS), of which there are three isoforms: neuronal NOS (nNOS/NOS1) and endothelial NOS (eNOS/NOS3) are expressed constitutively and require calcium for activation, while inducible NOS (iNOS/NOS2) is transcriptionally regulated, and thus has calcium-independent activity. NADPH-diaphorase activity and nNOS-like immunoreactivity can be found in all major cell types in the chick retina and choroid^39–41^ and are co-localized consistently in approximately 15 types of retinal neurons; they may be absent from RPE and scleral chondrocytes^40^. Localization of eNOS and iNOS in ocular tissues has been reported as widespread expression in the chick retina^41^, but these results may not be conclusive, as the authors were unable to reliably co-localize endogenously generated NO signal with eNOS- and iNOS-like immunoreactivity. As reported by us in the present paper, intravitreal delivery of NO sources (L-Arg and SNP) significantly inhibited FDM, while blockade of NO-synthesis prevented myopia-inhibition by atropine and L-Arg. These results support the role of nitric oxide as a “stop” signal in regulation of eye growth, and are in agreement with previous studies that either directly^25, 42^ (and Chakraborty *et al*. IOVS 2016; 57: E-Abstract 4742) or indirectly^21, 23^ link changes in ocular nitric oxide synthesis with differential effects on myopia.

**Figure 6:**
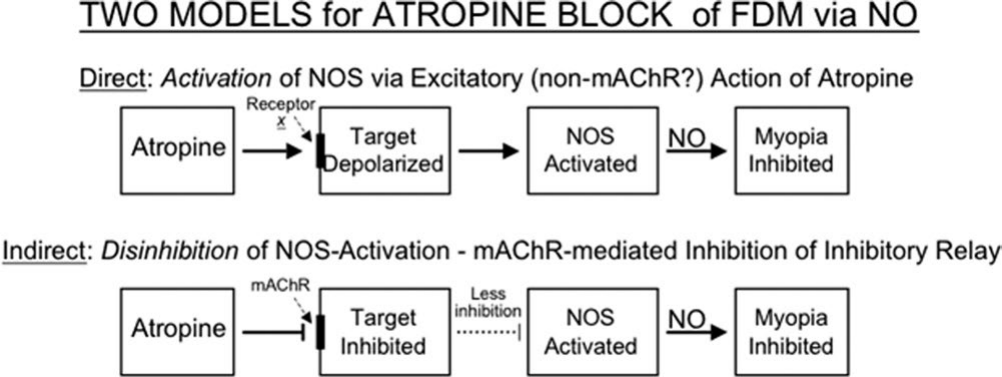
Two possible mechanisms for induction of NO synthesis by atropine, suggested by the results reported in this paper. TOP: In the direct pathway, atropine could cause *excitation* (depolarization) of a target cell; this would cause an increase in intracellular calcium, and possibly activation of nNOS or eNOS – thus leading to NO synthesis and release, and prevention of myopia. BOTTOM: In an indirect pathway, atropine could cause *inhibition* of a target cell that releases inhibitory transmitters – causing decreased release of neurotransmitter, and thus excitation of the cell that was being inhibited (disinhibition) – finally leading to an increase in intracellular calcium, activation of constitutively expressed nNOS or eNOS, induction of NO synthesis, and prevention of myopia. Each step could represent the overall function of more complex pathway.

Given the limited information currently available in the literature, we can only speculate as to the pathways and mechanisms by which atropine might induce NO synthesis in the retina (or other ocular tissues). Figure 6 outlines two possible scenarios, using mAChRs as an example. Direct Pathway/Excitation: If atropine *does* work via mAChRs, its targets would likely be M2/M4, which are G_i_-coupled and generally produce inhibitory effects when activated by ACh^43^. Here, muscarinic antagonism by atropine would cause cellular excitation and depolarization by blocking the constitutive inhibitory activity of the mAChR target(s). The resulting cellular excitation would in turn increase the concentration of intracellular calcium that can drive NO synthesis by stimulating constitutive nNOS or eNOS. Indirect Pathway/Disinhibition: If atropine were working via mAChR subtypes M1/M3/M5 it might increase NO concentrations indirectly, by disrupting inhibitory circuitry in the inner retina. Atropine would block its target receptor on an inhibitory interneuron, resulting in decreased release of the inhibitory neurotransmitter and depolarization of whatever cell was targeted by that interneuron – in this case, a cell containing NOS. Depolarization would lead to an increase in intracellular calcium and subsequent activation of eNOS or nNOS, resulting in myopia inhibition. It is important to note that chickens do not have an M1 subtype equivalent receptor^44^; instead, the chick M2-like receptor has a motif that gives it M1-like affinity for pirenzepine^45^.

Alternatively, we suggest the compelling hypothesis that it is not the interaction of atropine with mAChRs *per se* that is responsible for growth inhibition, but instead either interaction with off-target (i.e., non-mAChR) receptors, or atropine-induced release of signalling molecules in the retina, that ultimately cause retardation of ocular growth. A good case can be made for the latter scenario. Schwahn *et al*.^46^ have shown that the intravitreal injection of myopia-inhibiting concentrations of atropine results in a massive increase in retinal dopamine-release. In agreement with this, immunoreactive chick m4 receptors are expressed universally by retinal dopaminergic neurons (100% of neurons studied, n = 75)^30^. Increasing retinal dopamine synthesis and release is well known to have strong myopia-inhibiting effects^47, 48^, and apomorphine, a nonselective dopamine agonist, inhibits lens-induced and form-deprivation myopia^47, 49^. Co-administration of atropine plus apomorphine does not result in an increased effect, however, leading Schmid *et al*. to suggest that these drugs may work at different points in the same pathway^50^.

The mechanism through which dopamine prevents myopia is also unclear, but evidence suggests that it too may stimulate the synthesis and release of NO^42^ (and Moinul, *et al*. *IOVS* 2012; 53: E-Abstract 3434). Considering the evidence, atropine could act in the retina to inhibit myopia by causing the release of dopamine, which in turn stimulates the synthesis and release of NO. It remains to be determined how this release of dopamine and subsequent NO synthesis may inhibit ocular growth, but NO is known to subserve many functions in the retina. One of these – the regulation of cell-cell coupling via gap junctions – has been implicated in myopia-inhibition by studies in our group (Teves, *et al*. *IOVS* 2014; 55: E-Abstract 3036). This type of signalling pathway could provide an explanation for some of the curiosities of the relationship between ACh, mAChRs, and eye growth. For example, it could explain why no significant changes are seen in mAChR expression and regulation during induction of FDM^31^, and why destroying >90% of ChAT-positive cells and enzyme activity (leaving dopaminergic amacrine cells intact) has little effect on the eye’s ability to grow normally or respond to form-deprivation and atropine-treatment^33^.

The existing evidence leaves no question that atropine prevents experimentally-induced myopia in chicks. Our data have confirmed this effect, while supporting for the first time an important role for NO in this process. The retina is the most likely candidate target tissue for atropine-mediated effects on myopia; it contains many kinds of cells and receptors with which atropine could interact, and it is recognized as the visual processing powerhouse of the eye, which drives the regulation of eye growth^28^. It is less likely that atropine would have a strong effect on either the choroid or the sclera, because of loss by diffusion, binding in the vitreous and retina, and limited penetration of the blood-retina barrier formed by the RPE. In addition, form-deprivation myopia is mediated by changes in function in local retinal circuits^51^, which cause local changes in signalling. However, the retina is not the only possible site of atropine’s anti-myopia action. Some evidence is consistent with an extra-retinal action of atropine^33, 52^ – with the choroid being a likely alternative target. The choroid relays growth-regulating signals from RPE to sclera^28, 50^ and it too contains cells which express mAChRs^30, 31^ and NOS^40^. Furthermore, the requirement of such a high dose of atropine to inhibit myopia is consistent with the limited ability of drugs to pass from vitreous to choroid.

More work is required to determine the mechanism by which atropine can prevent myopia, but its dependence on nitric oxide is an important clue, suggesting possible new treatment options. Targeting NO instead of mAChR mechanisms would allow us to control human myopia without the M3-mediated side effects of photophobia, glare, and loss of accommodation, and might allow us to use more dilute drug concentrations, alleviating allergic side-effects. Light-therapy may be a better option, as it is linked to an increase in ocular NO^17, 18^ and protection against myopia development^23, 24^. This approach would not require special equipment or expose the subject to the potentially serious side-effects of pharmaceuticals. Thus, the focus on NO as the therapeutic mediator could lead to a paradigm shift in the treatment of myopia.

### Study Limitations

A-scan ultrasound would have allowed us to measure the relevant internal axial dimensions of the eye – such as vitreous chamber depth, which is more informative than overall eye length, or choroidal thickness, which has been correlated with changes in myopia development due to treatment with muscarinic drugs^52^ and nitric oxide synthase inhibitors^25, 53^. It would be useful to compare such results with those in previous studies, which utilized a different NOS inhibitor (L-NAME) whose mechanism of action may differ from that of L-NMMA or L-NIO^54^; unfortunately, high-resolution ultrasonography was not available to us during the course of this study. The data derived by our methods are reliable, however, and clearly demonstrate significant effects relevant to the primary concern of this study. Myopia development was significantly attenuated by NO-sources and atropine, and FDM-inhibition by atropine was prevented completely by addition of specific NOS-inhibitors.

## Materials and Methods

### Ethics & Animals

Animal use protocols were approved by the Health Sciences Animal Care Committee of the University of Calgary (Protocol #M10008 & AC14-0134), and were carried out in accordance with the CCAC Guide to the Care and Use of Experimental Animals and the ARVO Statement for the Use of Animals in Ophthalmic and Vision Research. White Leghorn cockerels (*Gallus gallus domesticus*, Shaver strain) were purchased from Rochester Hatchery (Westlock, Alberta, Canada) and delivered on post-hatching day one (P1). They were housed at the University of Calgary Health Sciences Animal Resource Centre (HSARC) at 26 °C, on a 12:12 light-dark schedule (lights on at 06:00), and given chick chow and water *ad libitum*. Mean illuminance in the housing and lab areas was 350–500 lux, provided by conventional indoor fluorescent lamps. At the end of experiments, chicks were euthanized by intraperitoneal injection of 0.2 mL Euthanyl (pentobarbital sodium, 240 mg/mL; CDMV, Saint-Hyacinthe, PQ, Canada), followed by decapitation.

### Drugs for Intravitreal Injection

Drugs, commercial sources, and the molar amounts delivered per injection are listed in Table 3. Drugs were dissolved in phosphate-buffered saline (PBS) (Gibco 14190-144; ThermoFischer Scientific) at room temperature. Stock solutions were made fresh on injection day one, and aliquots were quick-frozen, stored at −20 °C, and used only for the duration of one experiment (six days) so as to avoid loss of drug activity due to prolonged storage. The concentrations tested were based on previously published data^25, 42, 54^ and preliminary results from our own dose-response studies in this specific strain of chick.

**Table 3:** Agents employed in the present studies.

Drug	Source	Cat#	Amt/Injection (Syringe)
Atropine Sulfate	Sigma-Aldrich	A0257	240 nmoles
L-arginine·HCl	Sigma-Aldrich	A5006	60–6000 nmoles
D-arginine·HCl	Sigma-Aldrich	A2646	10 µmoles
L-NIO	Sigma-Aldrich	I134	6 nmoles
L-NMMA (acetate)	Cayman Chemicals	10005031	6 nmoles
D-NMMA (acetate)	Cayman Chemicals	14186	6 nmoles
Sodium nitroprusside	Sigma-Aldrich	71778	10–1000 nmoles

### Rationale for Drug Selection

L-arginine (L-Arg) increases intraocular NO by supplementing the endogenous L-Arg substrate for NOS, thus boosting NO-synthesis at local sources; in contrast, sodium nitroprusside (SNP) releases NO spontaneously and diffusely, without restriction to sites of local NO synthesis, release, and action. The best-known effect of these drugs is vasodilation caused by NO-activation of soluble guanylyl cyclase and subsequent cGMP-mediated smooth muscle relaxation^55, 56^. However, we do not yet know enough about the role of NO in the retina to say whether these vasodilatory effects might underlie myopia prevention; it is not likely for the chick model, as the avian retina is avascular. N^G^-(1-Iminoethyl)-L-ornithine (L-NIO), and L-N^G^-monomethyl arginine (L-NMMA) are analogues of L-Arg, which (unlike L-NAME) are transported readily into nitroxergic cells by the L-Arg transporter, bind to the active site of NOS, and competitively inhibit L-Arg binding and NO synthesis^54^. D-N^G^-monomethyl arginine (D-NMMA), an enantiomer of L-NMMA, is arginine-like but has insignificant NOS-inhibiting activity, and is a negative control for the stereospecific action of L-NMMA at NOS; D-arginine serves a similar experimental role, acting as a negative control for L-Arg.

### Induction of Myopia, and Intravitreal Injections

Form-deprivation myopia (FDM) was induced, starting on days P7-P8, by affixing translucent diffuser goggles over the right eye (OD) using contact cement; the left eye (OS) was a non-occluded, vehicle-injected, within-animal control. Goggles remained in place during injections; small triangular vents in the top of the goggles provided needle access and promoted air circulation, without significantly diminishing the form-deprivation effect. Beginning one day after goggle application, intravitreal injections were performed at the same time each day (11:00 am–12:30 pm), every other day, in order to minimize the chicks’ discomfort and any growth-retarding effects of needle-puncture^57^. Chicks were anesthetized with 1.5% isofluorane in O_2_:N_2_O (50:50), the upper eyelids were cleaned externally with 70% ethanol, and drugs were injected using a 26 gauge needle on a 25 µL Hamilton Gastight syringe. The needle was inserted approximately 6 mm deep, through eyelid and sclera, into the dorsal quadrant of the eye, and 20 µL was injected rapidly into the vitreous. The needle was then slowly withdrawn and rinsed in 70% ethanol before the next injection of the same drug solution; dedicated syringes and needles were used for each drug. Since the vitreous chamber volume in these chicks is about 200 µl (W.K. Stell & D. Rushforth, 2002, unpublished studies), the maximum drug concentration that could be presented to the retina after injection (assuming uniform diffusion throughout the vitreous) was roughly 10% of the concentration injected. The same injection site was not used for subsequent injections, to minimize local scarring and backflow through the injection holes. After injections, chicks were returned to their cage to recover.

### Measurements

The day after the final injection, refractive error (±0.5 D) was measured without cycloplegia using a streak retinoscope (Model 18100; WelchAllyn Canada) and trial lenses; working distance was approximately 0.5 m, and no correction was made for distance or the small-eye artefact. Subsequently, the chicks were euthanized, the eyes were removed, and tissues on the outer surface of the globe were dissected away. The globe was placed in a Petri dish supported by a PBS-dampened paper towel, for viewing either perpendicular to the optic axis (for axial length) or on-axis with the corneal side up (for equatorial diameter). *Axial length* was defined as the distance from front of cornea to back of sclera; *equatorial diameter* of these ellipsoidal eyes was defined as the mean of the maximum and minimum diameters ([d_**max**_ + d_**min**_]/2). Measurements of the globe (±0.01 mm) were made with digital calipers (Model 58-6800-4; Mastercraft); while viewing both globe and calipers under a dissecting microscope, the caliper arms were closed until they just barely touched the surfaces of the eye. Wet weight of the eye (±0.001 g) was measured using a digital balance (PL200; Mettler). Measurement bias was minimized by blinding the experimenter to the chick’s treatment, and by recording refractive error separately from biometric parameters. Recorded values were the average of three separate measurements, which typically were completed within 30 seconds per eye; therefore, any changes in dimensions or weight due to handling and drying were negligible.

### Data Analysis

Drug treatments were found not to affect control eyes (see Results); therefore, the effects of treatment are expressed as the mean difference between values for experimental-eye (goggled, drug-injected) and control-eye (open, vehicle-injected), ±standard deviation; data for PBS- and atropine-injected eyes were pooled separately for final analysis. Data for the treatment groups were analysed using One-Way ANOVA with Tukey’s or Dunn’s post hoc test (Prism V6.02; GraphPad Software, Inc., LaJolla CA, USA), unless specified otherwise, and were deemed significant at p < 0.05.

### Tissue preparation, Histology, and Immunolabelling

Eyes were hemisected through the equator, vitreous was removed, and posterior eye cups were fixed in 4% paraformaldehyde + 3% sucrose in 0.1 M phosphate buffer, pH 7.4, for 0.5 hrs (25 °C), then washed three times (15 min each) in PBS, and cryoprotected in 0.1 M phosphate buffer + 30% sucrose for 2–4 days at 4 °C. For cryosectioning, eye cups were soaked in OCT (VWR) for 15 min (25 °C) and quick-frozen using dry ice/ethanol. Sections were cut at 12–14 µm, thaw-mounted onto Fisherbrand Superfrost Plus slides (ThermoFischer Scientific), briefly heat-fixed on a hot plate (40 °C, 10 min), ringed with rubber cement to create a hydrophobic barrier, and stored at −20 °C.

For histology, slides were warmed (25 °C), washed three times (15 min) in PBS, and incubated under 0.1% (w/v) Toluidine blue^58^. After 2 min, the stain was removed, and the slides were washed in PBS and mounted under cover-slips in a 4:1 solution of glycerol:water. For detecting signs of damage by immunolabelling, we used two monoclonal mouse antibodies (both known to be specific for application to chicken tissues) from the Developmental Studies Hybridoma Bank (DSHB; University of Iowa, Ames, IA, USA): anti-LEP-100 (lysosomal membrane glycoprotein, cv24)^59^ and anti-GRL2 (activated leukocyte cell-surface glycoprotein GRL2)^29^, which are markers for microglia/macrophages^26, 60^ and activated phagocytes/granulocytes^61^, respectively. Sections were warmed, washed, and then incubated overnight at 25 °C in LEP-100 (1:50) or GRL2 (1:500) antibodies, diluted in PBS + 0.025% Triton X-100. After incubation, the slides were washed in PBS and then incubated under 1:1000 AF488 donkey anti-mouse secondary antibody (Jackson ImmunoResearch Laboratories, Inc., West Grove, PA, USA) for 2 hrs. Slides were washed again in PBS, and mounted using Fluoroshield mounting medium + DAPI (Abcam Inc., Toronto, ON, Canada). Toluidine blue-labeled slides were imaged using a Zeiss epi-illumination microscope with 25x Neo-Fluar water-immersion objective, NA = 0.8, and digital camera (Model RT3; SPOT Imaging, Division of Diagnostic Instruments, Inc., Sterling Heights, Michigan, USA). LEP-100- and GRL2-labeled slides were imaged using a laser-scanning confocal microscope with 40x oil-immersion objective, NA = 1.3 (Model FV1000; Olympus Corporation of the Americas, Center Valley, PA, USA). Image post-processing was performed using Adobe Photoshop CS5 (Adobe Systems Incorporated, San Jose, CA, USA).

## Electronic supplementary material

Supplementary Information

## References

[1] Vitale S, Sperduto RD, Ferris FL (2009). Increased prevalence of myopia in the United States between 1971–1972 and 1999–2004. Arch Ophthalmol.

[2] Foster PJ, Jiang Y (2014). Epidemiology of myopia. Eye (Lond).

[3] Shih KC (2016). Use of Atropine for Prevention of Childhood Myopia Progression in Clinical Practice. Eye Contact Lens.

[4] Chia A (2012). Atropine for the treatment of childhood myopia: safety and efficacy of 0.5%, 0.1%, and 0.01% doses (Atropine for the Treatment of Myopia 2). Ophthalmology.

[5] Chua WH (2006). Atropine for the treatment of childhood myopia. Ophthalmology.

[6] Chia A (2014). Atropine for the treatment of childhood myopia: changes after stopping atropine 0.01%, 0.1% and 0.5%. Am J Ophthalmol.

[7] Siatkowski RM (2008). Two-year multicenter, randomized, double-masked, placebo-controlled, parallel safety and efficacy study of 2% pirenzepine ophthalmic gel in children with myopia. J aapos.

[8] Manny RE (2001). Tropicamide (1%): an effective cycloplegic agent for myopic children. Invest Ophthalmol Vis Sci.

[9] Luft WA, Ming Y, Stell WK (2003). Variable effects of previously untested muscarinic receptor antagonists on experimental myopia. Invest Ophthalmol Vis Sci.

[10] Leech EM, Cottriall CL, McBrien NA (1995). Pirenzepine prevents form deprivation myopia in a dose dependent manner. Ophthalmic Physiol Opt.

[11] Rickers M, Schaeffel F (1995). Dose-dependent effects of intravitreal pirenzepine on deprivation myopia and lens-induced refractive errors in chickens. Exp Eye Res.

[12] McBrien NA, Moghaddam HO, Reeder AP (1993). Atropine reduces experimental myopia and eye enlargement via a nonaccommodative mechanism. Invest Ophthalmol Vis Sci.

[13] Noll GN, Billek M, Pietruck C, Schmidt KF (1994). Inhibition of nitric oxide synthase alters light responses and dark voltage of amphibian photoreceptors. Neuropharmacology.

[14] Tsuyama Y, Noll GN, Schmidt KF (1993). L-arginine and nicotinamide adenine dinucleotide phosphate alter dark voltage and accelerate light response recovery in isolated retinal rods of the frog (Rana temporaria). Neurosci Lett.

[15] Shiells R, Falk G (1992). Retinal on-bipolar cells contain a nitric oxide-sensitive guanylate cyclase. Neuroreport.

[16] Sato M, Ohtsuka T, Stell WK (2011). Endogenous nitric oxide enhances the light-response of cones during light-adaptation in the rat retina. Vision Res.

[17] Hoshi H, Sato M, Oguri M, Ohtsuka T (2003). In vivo nitric oxide concentration in the vitreous of rat eye. Neurosci Lett.

[18] Neal M, Cunningham J, Matthews K (1998). Selective release of nitric oxide from retinal amacrine and bipolar cells. Invest Ophthalmol Vis Sci.

[19] Wellard JW, Morgan IG (1996). Nitric oxide donors mimic the effects of light on photoreceptor melatonin synthesis. Aust N Z J Ophthalmol.

[20] Shi, Q. Mechanisms of Adaptation to Mean Light Intensity in the Chick Retina. PhD thesis, Neuroscience, University of Calgary (2014).

[21] Ashby R, Ohlendorf A, Schaeffel F (2009). The effect of ambient illuminance on the development of deprivation myopia in chicks. Invest Ophthalmol Vis Sci.

[22] Wang Y (2015). Exposure to sunlight reduces the risk of myopia in rhesus monkeys. PLoS One.

[23] Rose KA (2008). Outdoor activity reduces the prevalence of myopia in children. Ophthalmology.

[24] French AN, Ashby RS, Morgan IG, Rose KA (2013). Time outdoors and the prevention of myopia. Exp Eye Res.

[25] Nickla DL, Wilken E, Lytle G, Yom S, Mertz J (2006). Inhibiting the transient choroidal thickening response using the nitric oxide synthase inhibitor l-NAME prevents the ameliorative effects of visual experience on ocular growth in two different visual paradigms. Exp Eye Res.

[26] Fischer AJ, Scott MA, Zelinka C, Sherwood P (2010). A novel type of glial cell in the retina is stimulated by insulin-like growth factor 1 and may exacerbate damage to neurons and Muller glia. Glia.

[27] Moretti M (2014). Role of agmatine in neurogenerative diseases and epilepsy in. Front Biosci (Elite Ed).

[28] Wallman J, Winawer J (2004). Homeostasis of eye growth and the question of myopia. Neuron.

[29] Thomas JL, Pourquie O, Coltey M, Vaigot P, Le Douarin NM (1993). Identification in the chicken of GRL1 and GRL2: two granule proteins expressed on the surface of activated leukocytes. Exp Cell Res.

[30] Fischer AJ, McKinnon LA, Nathanson NM, Stell WK (1998). Identification and localization of muscarinic acetylcholine receptors in the ocular tissues of the chick. J Comp Neurol.

[31] Vessey KA, Cottriall CL, McBrien NA (2002). Muscarinic receptor protein expression in the ocular tissues of the chick during normal and myopic eye development. Brain Res Dev Brain Res.

[32] McBrien NA, Cottriall CL, Annies R (2001). Retinal acetylcholine content in normal and myopic eyes: a role in ocular growth control?. Vis Neurosci.

[33] Fischer AJ, Miethke P, Morgan IG, Stell WK (1998). Cholinergic amacrine cells are not required for the progression and atropine-mediated suppression of form-deprivation myopia. Brain Res.

[34] McBrien NA, Stell WK, Carr B (2013). How does atropine exert its anti-myopia effects?. Ophthalmic Physiol Opt.

[35] Cimini BA, Strang CE, Wotring VE, Keyser KT, Eldred WD (2008). Role of acetylcholine in nitric oxide production in the salamander retina. J Comp Neurol.

[36] Borda E, Berra A, Saravia M, Ganzinelli S, Sterin-Borda L (2005). Correlations between neuronal nitric oxide synthase and muscarinic M3/M1 receptors in the rat retina. Exp Eye Res.

[37] Neal M, Cunningham J, Matthews K (1997). Nitric oxide enhancement of cholinergic amacrine activity by inhibition of glycine release. Invest Ophthalmol Vis Sci.

[38] Okada M, Osumi Y, Okuma Y, Ueno H (2001). Nitric oxide inhibits the release of acetylcholine in the isolated retina. Graefes Arch Clin Exp Ophthalmol.

[39] Wilson M, Nacsa N, Hart NS, Weller C, Vaney DI (2011). Regional distribution of nitrergic neurons in the inner retina of the chicken. Vis Neurosci.

[40] Fischer AJ, Stell WK (1999). Nitric oxide synthase-containing cells in the retina, pigmented epithelium, choroid, and sclera of the chick eye. J Comp Neurol.

[41] Tekmen-Clark M, Gleason E (2013). Nitric oxide production and the expression of two nitric oxide synthases in the avian retina. Vis Neurosci.

[42] Nickla DL, Lee L, Totonelly K (2013). Nitric oxide synthase inhibitors prevent the growth-inhibiting effects of quinpirole. Optom Vis Sci.

[43] Migeon JC, Nathanson NM (1994). Differential regulation of cAMP-mediated gene transcription by m1 and m4 muscarinic acetylcholine receptors. Preferential coupling of m4 receptors to Gi alpha-2. J Biol Chem.

[44] Yin GC, Gentle A, McBrien NA (2004). Muscarinic antagonist control of myopia: a molecular search for the M1 receptor in chick. Mol Vis.

[45] Tietje KM, Nathanson NM (1991). Embryonic chick heart expresses multiple muscarinic acetylcholine receptor subtypes. Isolation and characterization of a gene encoding a novel m2 muscarinic acetylcholine receptor with high affinity for pirenzepine. J Biol Chem.

[46] Schwahn HN, Kaymak H, Schaeffel F (2000). Effects of atropine on refractive development, dopamine release, and slow retinal potentials in the chick. Vis Neurosci.

[47] Rohrer B, Spira AW, Stell WK (1993). Apomorphine blocks form-deprivation myopia in chickens by a dopamine D2-receptor mechanism acting in retina or pigmented epithelium. Vis Neurosci.

[48] Stone RA, Lin T, Laties AM, Iuvone PM (1989). Retinal dopamine and form-deprivation myopia. Proc Natl Acad Sci USA.

[49] Nickla DL, Totonelly K, Dhillon B (2010). Dopaminergic agonists that result in ocular growth inhibition also elicit transient increases in choroidal thickness in chicks. Exp Eye Res.

[50] Schmid KL, Wildsoet CF (2004). Inhibitory effects of apomorphine and atropine and their combination on myopia in chicks. Optom Vis Sci.

[51] Wallman J, Gottlieb MD, Rajaram V, Fugate-Wentzek LA (1987). Local retinal regions control local eye growth and myopia. Science.

[52] Nickla DL, Zhu X, Wallman J (2013). Effects of muscarinic agents on chick choroids in intact eyes and eyecups: evidence for a muscarinic mechanism in choroidal thinning. Ophthalmic Physiol Opt.

[53] Nickla DL, Wildsoet CF (2004). The effect of the nonspecific nitric oxide synthase inhibitor NG-nitro-L-arginine methyl ester on the choroidal compensatory response to myopic defocus in chickens. Optom Vis Sci.

[54] Wellard JW, Miethke P, Morgan IG (1995). Neural barriers affect the action of nitric oxide synthase inhibitors in the intact chicken retina. Neurosci Lett.

[55] Martin W, Furchgott RF, Villani GM, Jothianandan D (1986). Phosphodiesterase inhibitors induce endothelium-dependent relaxation of rat and rabbit aorta by potentiating the effects of spontaneously released endothelium-derived relaxing factor. J Pharmacol Exp Ther.

[56] Palmer RM, Ferrige AG, Moncada S (1987). Nitric oxide release accounts for the biological activity of endothelium-derived relaxing factor. Nature.

[57] Rohrer B, Iuvone PM, Stell WK (1995). Stimulation of dopaminergic amacrine cells by stroboscopic illumination or fibroblast growth factor (bFGF, FGF-2) injections: possible roles in prevention of form-deprivation myopia in the chick. Brain Res.

[58] Fischer AJ, Morgan IG, Stell WK (1999). Colchicine causes excessive ocular growth and myopia in chicks. Vision Res.

[59] Fambrough DM, Takeyasu K, Lippincott-Schwarz J, Siegel NR (1988). Structure of LEP100, a glycoprotein that shuttles between lysosomes and the plasma membrane, deduced from the nucleotide sequence of the encoding cDNA. J Cell Biol.

[60] Fischer AJ, Seltner RL, Poon J, Stell WK (1998). Immunocytochemical characterization of quisqualic acid- and N-methyl-D-aspartate-induced excitotoxicity in the retina of chicks. J Comp Neurol.

[61] Thomas JL, Stieber A, Gonatas N (1994). Two proteins associated with secretory granule membranes identified in chicken regulated secretory cells. J Cell Sci.

