# Supplementary material for: Nitric Oxide (NO) Mediates the Inhibition of Form-Deprivation Myopia by Atropine in Chicks
Source: Sci Rep. 2016 Dec 5;6:9. doi: 10.1038/s41598-016-0002-7 (PMC5431363; doi:10.1038/s41598-016-0002-7)
Supplement: Supplementary file 1 — Supplementary Information [file 41598_2016_2_MOESM1_ESM.pdf]

Nitric Oxide (NO) Mediates the Inhibition of Form-Deprivation Myopia by Atropine in Chicks

Brittany J. Carr[1] and *William K. Stell[2]

[1]Neuroscience Graduate Program, [1]Snyder Institute for Chronic Diseases, [1,2]Alberta Children's Hospital Research Institute, [1,2]Hotchkiss Brain Institute, and [2]Department of Cell Biology and Anatomy, and Department of Surgery; Cumming School of Medicine, University of Calgary, Calgary, Alberta, Canada.

Supplementary Figures: 4

Communicating Authors: *William K. Stell, Department of Cell Biology and Anatomy, Cumming School of Medicine, University of Calgary, 3330 Hospital Dr. NW, Calgary, AB, Canada T2N 4N1; (tel) +1-403-220-7501; (email) wstell@ucalgary.ca.

Brittany J. Carr, Department of Neuroscience, Cumming School of Medicine, University of Calgary, 3330 Hospital Dr. NW, Calgary, AB, Canada T2N 4N1; (tel) +1-403-220-8724; (email) bcarr@ucalgary.ca.

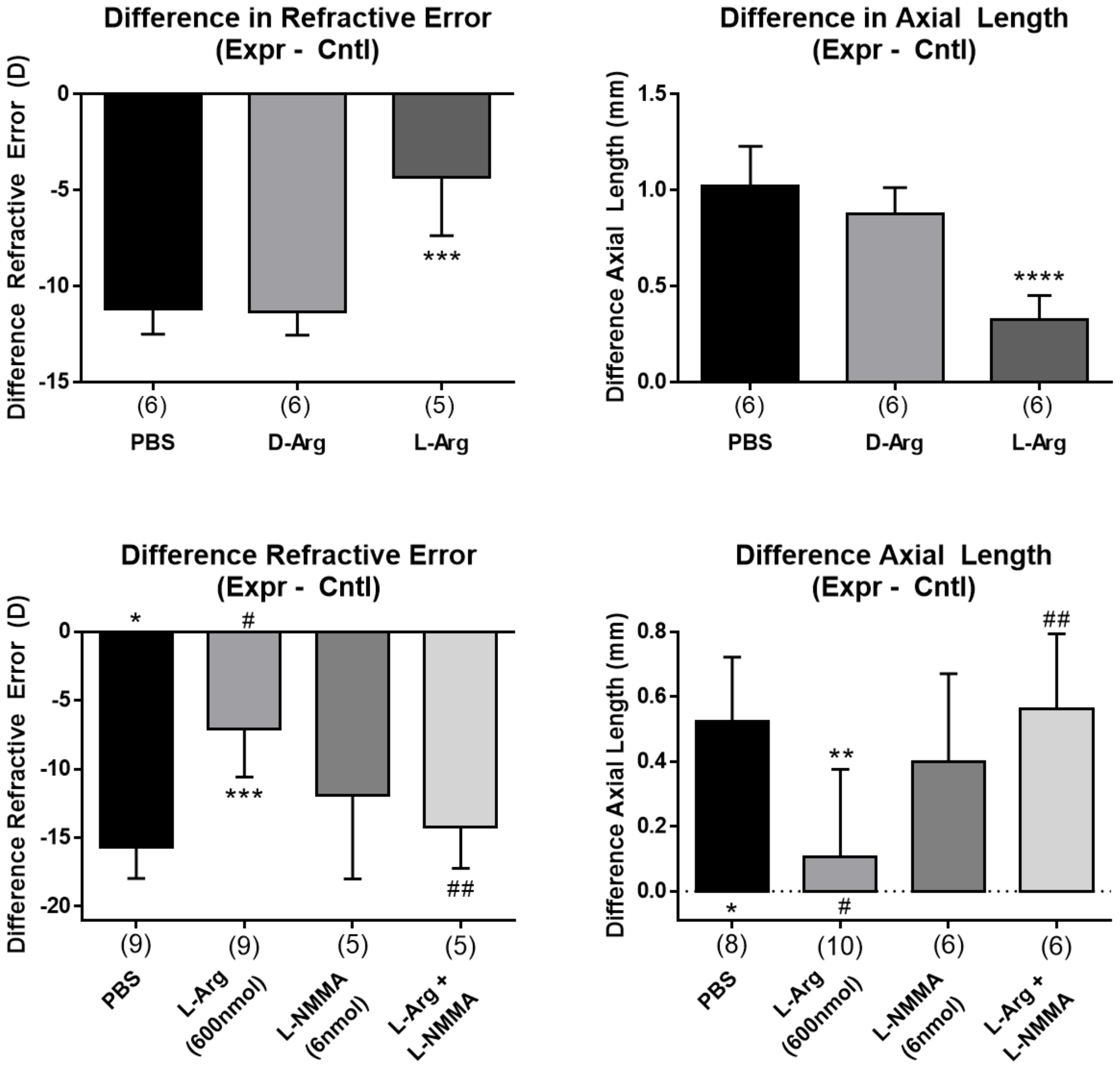


**Supplementary Figure S1**. TOP: Effect of D-Arginine (D-Arg: 10μmol) or L-Arginine (L-Arg: 600nmol) on the differences in refractive error and axial length induced during FDM; L-Arg successfully inhibits FDM, while D-Arg has no effect. BOTTOM: Effect of L-NMMA (6nmol) on FDM-inhibition by L-Arg (600nmol); simultaneous injection of L-NMMA with L-Arg abolishes myopia inhibition. ***Symbols:*** asterisk (*): comparison to effect of PBS-treatment; pound (#): comparison to L-Arg-treatment. ***Statistics:*** **••••** p<0.0001, **•••** p<0.0001, **••** p<0.01; TOP: Kruskal-Wallis + Dunn's post-hoc; BOTTOM: One-Way ANOVA + Tukey's post-hoc. Data are represented as the means of the difference in values for the experimental eye minus those for the control eye, ± SD; sample sizes (n) are denoted in brackets below each column.

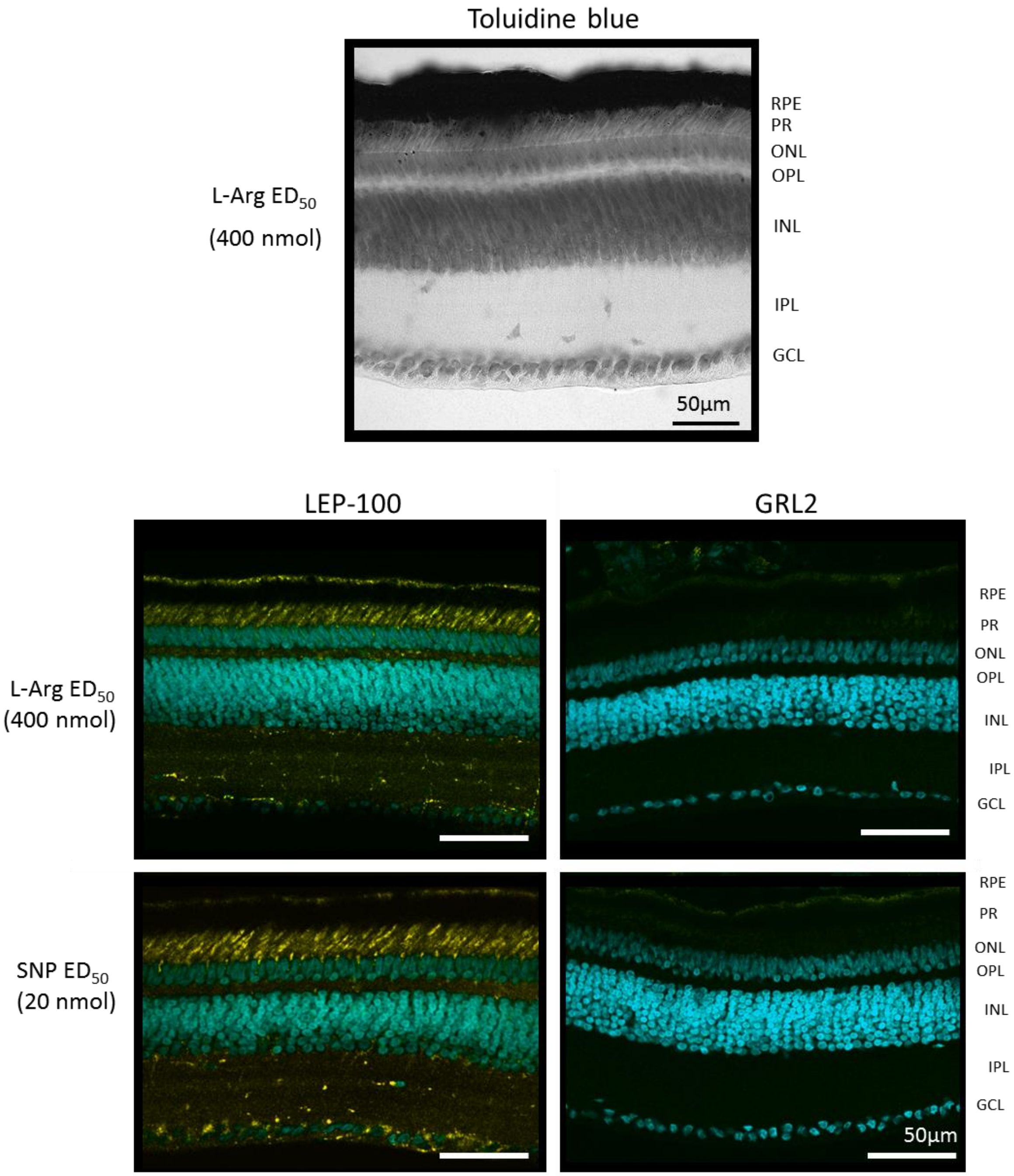


**Supplementary Figure S2**. TOP: Toludine blue labelling of retina treated with $ED_{50}$ dose of L-Arginine (L-Arg: 400nmol/injection); histology does not differ significantly from that at the highest dose of L-Arg or PBS controls (main text, Fig. 3). Widefield (25x, NA 0.8). Scale bar = 50µm. BOTTOM: LEP-100 (left) and GRL2 (right) labeling of retinas treated with $ED_{50}$ doses of L-Arg (400nmol/injection) and SNP (20nmol/injection). Labeling did not differ significantly between treatment groups, or from PBS controls (main text, Fig. 4). Yellow-green: LEP-100 or GRL2, cyan: DAPI. Pictures are representative of a single confocal plane (40x, NA 1.3, z-thickness=1.096µm). Scale bar = 50µm.

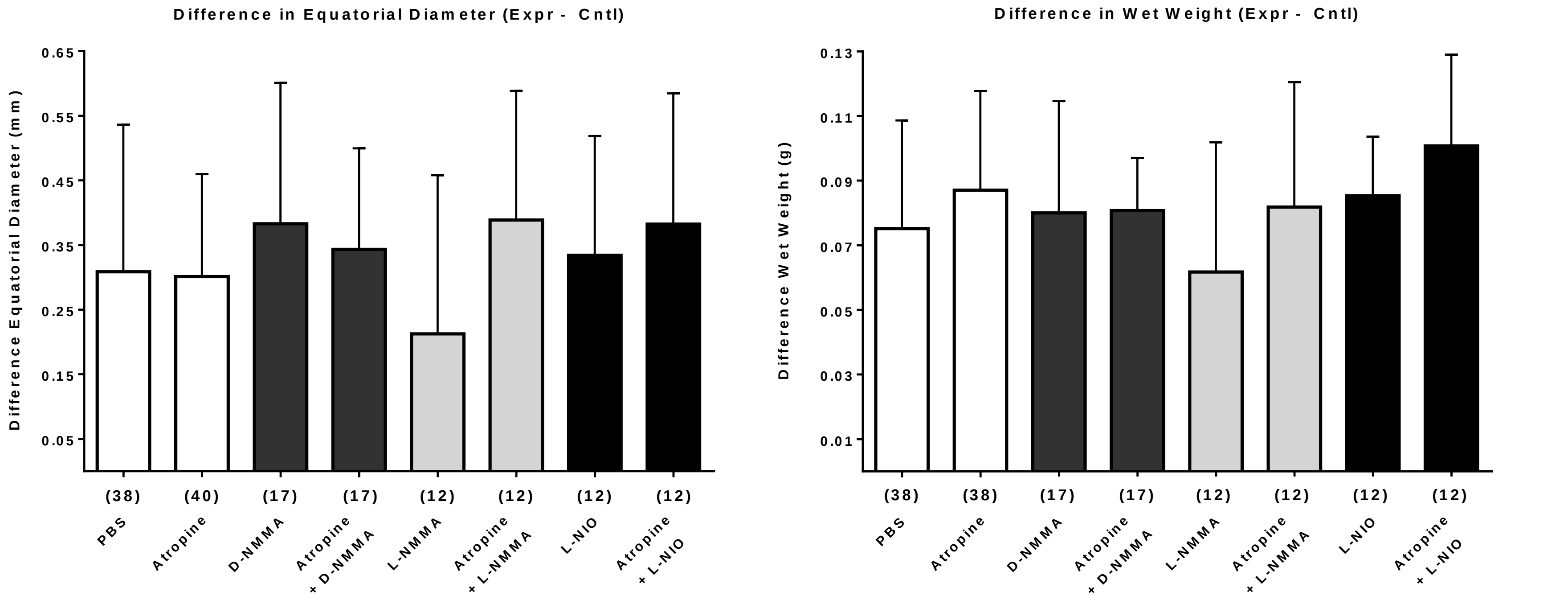


**Supplementary Figure S3.** Effects of atropine (240nmol), NOS inhibitors (6nmol; L-NIO, L-NMMA), D-NMMA (6nmol), and the combination of atropine + NOS inhibitors (L-NIO, L-NMMA), or atropine + D-NMMA on equatorial diameter and wet weight. These data did not follow the same trends as the data for difference in refractive error and difference in axial length (main text, Fig 5), and there was no significant effect of any treatment on these parameters (One-Way ANOVA). ***Abbreviations***: **L-NIO** [$N^G$-(1-Iminoethyl)-L-ornithine]; **L-NMMA** [L-$N^G$-monomethyl arginine]; **D-NMMA** [D-$N^G$-monomethyl arginine]. Data are represented as the means of the difference in values for the experimental eye minus those for the control eye, ± SD; sample sizes (n) are denoted in brackets below each column.

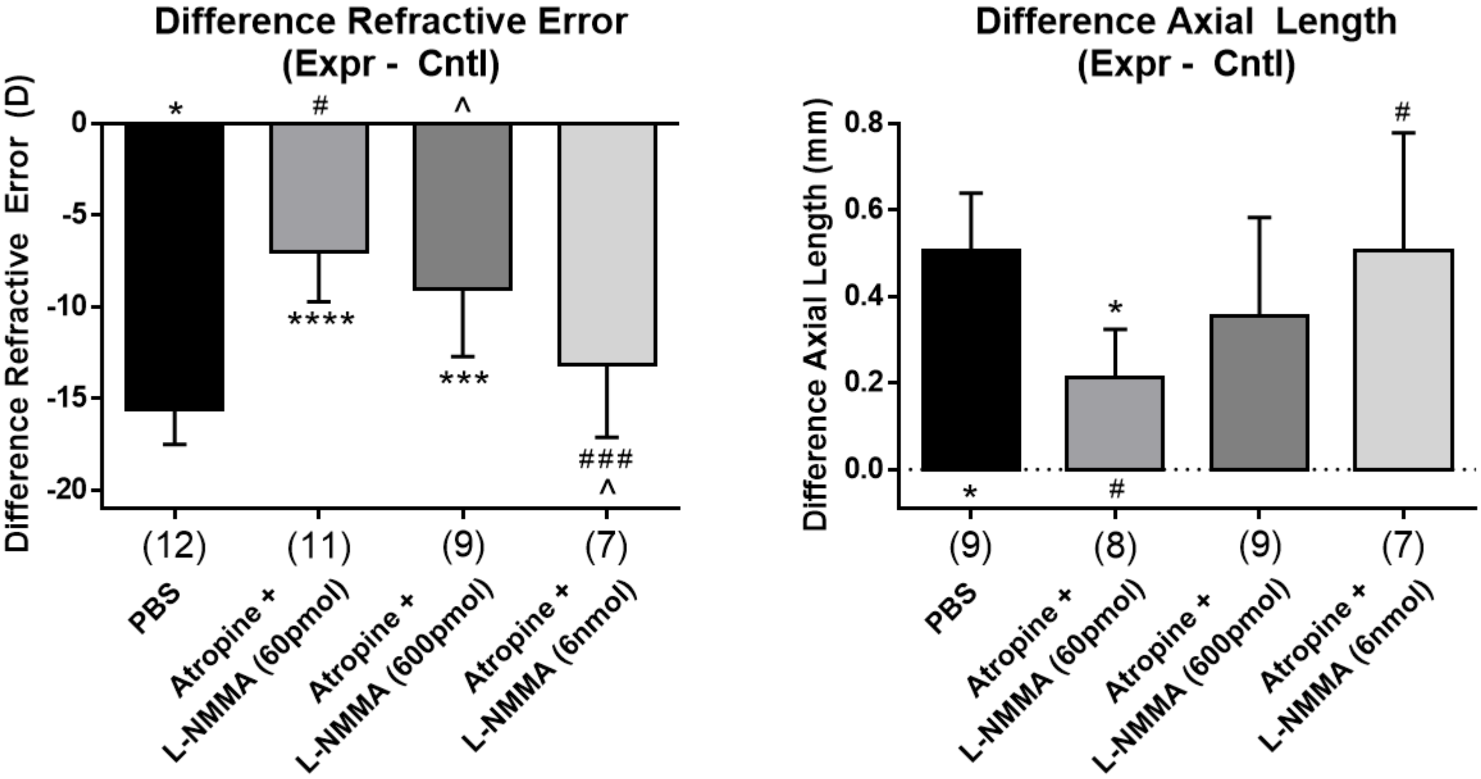


**Supplementary Figure S4**. Dose-dependent effects of L-NMMA on atropine-mediated myopia inhibition. As the concentration of L-NMMA is decreased from 6nmol to 60pmol, its ability to block myopia inhibition by atropine (240nmol) is lost. ***Abbreviations***: **L-NMMA** [L-$N^G$-monomethyl arginine]. ***Symbols:*** asterisk (*): comparison to effect of PBS-treatment; pound (#): comparison to 60pmol; caret (^): comparison to 600pmol. ***Statistics:*** •••• p<0.0001, ••• p<0.0001, • p<0.05; One-Way ANOVA + Tukey's post-hoc. Data are represented as the means of the difference in values for the experimental eye minus those for the control eye, ± SD; sample sizes (n) are denoted in brackets below each column.
